# Re-evaluating the driving force behind mutations

**DOI:** 10.7554/eLife.89706

**Published:** 2023-10-11

**Authors:** Thibault Leroy

**Affiliations:** 1 GenPhySE, INRAE, INP, ENVT, Université de Toulouse Auzeville-Tolosane France

**Keywords:** heritable mutations, tropical trees, plants, evolution, Other

## Abstract

Experiments on tropical trees suggest that new mutations in plants are driven by age rather than number of cell divisions during growth.

**Related research article** Satake A, Imai R, Fujino T, Tomimoto S, Ohta K, Na’iem M, Indrioko S, Widiyatno, Purnomo S, Mollá–Morales A, Nizhynska V, Tani N, Suyama Y, Sasaki E, Kasahara M. 2023. Somatic mutation rates scale with time not growth rate in long-lived tropical trees. *eLife* **12**:RP88456. doi: 10.7554/eLife.88456.

Despite the important role they play in our environment, plants are often perceived to be less complex than animals, particularly in regards to their functional and evolutionary processes (Jose et al., 2019). A fundamental question in evolution is how heritable mutations, which can be transmitted to future generations, accumulate in the genome. However, this question has been little explored to date in plants compared to animals.

In animals, it was initially assumed that mutations predominately came from errors during DNA replication, causing them to appear at the same rate as cell division. However, detailed investigations over the last decade have revealed that heritable mutations accumulate with age rather than with the number of cell divisions. This is supported by data showing that the maternal age at conception contributes to the number of new mutations passed to progeny, because oocytes do not divide after childhood (Figure 1; Goldmann et al., 2016; Jónsson et al., 2017). Consequently, it is now widely accepted that the rate animals acquire heritable mutations is mostly independent from replication, and instead driven by unrepaired DNA damage accumulating with age. This also explains why certain patterns of mutations are more common, such as a high proportion of cytosine-to-thymine mutations (Gao et al., 2019).

**Figure 1.**
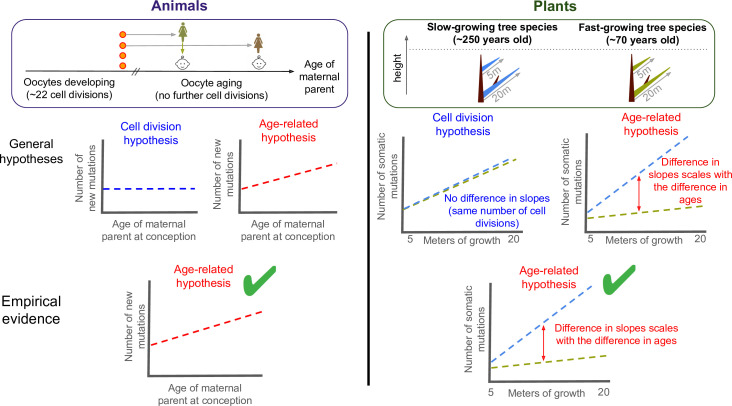
Testing what drives mutations in animals and plants. There are two hypotheses for how mutations appear and are putatively passed down to future offspring: through errors during DNA replication (cell division hypothesis), or unrepaired damage accumulating with age (age-related hypothesis). To test what drives germline mutations in animals (left panel), previous studies compared the age of the maternal parent at conception to the number of new mutations in the offspring of mammals. This revealed a positive correlation between the two variables (bottom graph, green tick). As oocytes stop dividing in childhood once they are fully formed, this suggests that heritable mutations are caused by age-related damage, not replication errors. Despite being typically harder to observe in males, heritable mutations transmitted from the paternal parent have also recently been shown to be consistent with the age-related hypothesis (Hahn et al., 2023). To test the two hypotheses in plants (right panel), Satake et al. calculated the number of somatic mutations per metre of growth in two evolutionary related tropical trees: a slow-growing (blue) and a fast-growing (green) species that were of similar heights but different ages. The two trees acquired somatic mutations at different rates (right graph), and the gap between these slopes corresponded to the age difference between them. This suggests that the age-related hypothesis also applies to plants (bottom panel, green tick), suggesting that there are parallels in how mutations arise in plants and animals, at least between mammals and trees.

Unlike animals, it is assumed that plants generally differentiate their germline late in development, although this remains debated (Lanfear, 2018). If this assumption is true, the mutations plants accumulate in their somatic, non-reproductive cells during growth will also be present in the germline and can be inherited by future generations. This intergenerational transmission is supported by empirical experiments in trees (Plomion et al., 2018; Wang et al., 2019; Schmitt et al., 2023). Mutation rates in plants are generally assumed to scale with the number of cell divisions in tissues as they grow, as well as UV exposure and other weakly supported general hypotheses (Schmitt et al., 2023). Now, in eLife, Akiko Satake from Kyushu University and colleagues report fascinating counter-intuitive evidence showing that aging rather than number of cell divisions appears to be the major driver of new somatic mutations in trees (Satake et al., 2023).

The team (who are based at various institutes in Japan, Indonesia and Austria) sequenced and assembled the genomes of two evolutionary related tropical trees living in central Borneo, Indonesia: a fast-growing species known as *Shorea leprosula,* and a slow-growing species known as *Shorea laevis*. Two individuals from each species were selected, which were of similar heights but different ages, with the *S. leprosula* tree being 66 years old and the *S. laevis* tree being 256 years old on average. DNA was extracted from the leaves at the ends of several branches and then compared to identify somatic mutations that were specific to each tree. This revealed that the slow-growing species had far more somatic mutations (962) than the faster-growing species (174).

If cell divisions drive mutations, one would expect similar mutation rates per meter of growth, after making reasonable assumptions for two evolutionary related species (Figure 1). Instead, Satake et al. found that the slow-growing species obtained 3.7 times more mutations per meter than the fast-growing tree, after considering the physical distance between branch tips. This value, however, is remarkably similar to the ratio between the average ages of the trees studied (256/66=3.9). These findings suggest that somatic mutations in plants are mostly driven by unrepaired damage that accumulates with age rather than replication-associated mutations.

Although the experimental design used by Satake et al. only identified a small fraction of the total number of somatic mutations, their results provide sufficient evidence to draw interesting parallels between plants and animals. Satake et al. also found additional evidence in support of this similarity that confirm previous reports: for instance, that the plant genome is enriched in cytosine-to-thymine mutations at specific positions, and shares mutation signatures with human cancers (Alexandrov et al., 2020). From a more methodological perspective, methods initially developed for cancer have been demonstrated to perform better for the discovery of somatic mutations in plants (Schmitt et al., 2022). Altogether, this suggests that mutational processes in plants and animals are largely conserved, and that plant and animal research communities have much to gain from collaborating with one another in the future.
